# Biomarkers of multi-organ dysfunction in ECMO: prognostic signposts for palliative transitions

**DOI:** 10.3389/fmed.2026.1762205

**Published:** 2026-01-21

**Authors:** Jia-Hao Zhang, Chor-Kuan Lim, Kuo-Fan Liao, Felisbela Gomes, Chen-Fen Tsai, Fu-Chien Hsieh

**Affiliations:** 1 Department of Nursing, Cardinal Tien College of Healthcare and Management, New Taipei, Taiwan; 2 Department of Critical Care Medicine, Far Eastern Memorial Hospital, Taipei, Taiwan; 3 Department of Chest Medicine, Far Eastern Memorial Hospital, Taipei, Taiwan; 4 Department of Respiratory and Internal Medicine, Regency Specialist Hospital, Masai, Johor, Malaysia; 5 Department of Traditional Chinese Medicine, Far Eastern Memorial Hospital, New Taipei City, Taiwan; 6 Institute of Clinical Medicine, National Yang Ming Chiao Tung University, Taipei, Taiwan; 7 Department of Nursing, Far Eastern Memorial Hospital, New Taipei City, Taiwan; 8 Division of Cardiovascular Surgery, Cardiovascular Center, Far Eastern Memorial Hospital, New Taipei City, Taiwan

**Keywords:** acute kidney injury, biomarkers, disseminated intravascular coagulation, extracorporeal membrane oxygenation, lactate, multi-organ dysfunction, palliative transitions, prognosis

## Abstract

Consistently high lactate levels and poor clearance on ECMO are important early signs of poor perfusion and useless care. For example, lactate levels over 8 mmol/L at 12 h, staying above 2.5 mmol/L at 24 h, or clearance rates below 22% are all strong signs that a patient will die in the hospital. Beyond metabolic markers, signs of specific organ failure are grave prognostic factors. Total bilirubin is a better way to check for liver problems than enzymes. A peak bilirubin level of more than 15 mg/dL means that the liver is not clearing the bilirubin properly and increases the risk of death by four times. Acute kidney injury requiring renal replacement therapy (CRRT) reflects severe multi-organ failure and is independently associated with mortality rates of 60–80%. Systemic coagulopathy also marks clinical decline, with an overt DIC score ≥5 or a precipitous ≥50% drop in platelets on the first day carrying an 8–9-fold increased risk of death. Furthermore, a profound inflammatory response, evidenced by high interleukin-6 and procalcitonin levels (≥0.5 μg/L), correlates with refractory shock and significantly worse outcomes compared to nonspecific markers like CRP. Ultimately, the persistence of these metabolic, hepatic, renal, and inflammatory abnormalities suggests irreversible organ damage and a poor prognosis.

## Highlights

- Lactate and Perfusion: Persistently elevated lactate levels and poor lactate clearance on ECMO indicate inadequate perfusion and portend high mortality. For example, lactate >8 mmol/L at 12 h or >2.5 mmol/L at 24 h after ECMO initiation strongly predicts in-hospital death. Patients with <22% lactate clearance by 12 h have nearly 3-fold higher hazard of mortality, underscoring that failure to normalize lactate is an early warning of futility.
- Hepatic Markers: Markers of liver injury, especially bilirubin, are critical prognostic indicators. ECMO patients who develop “shock liver” (e.g., AST/ALT >20 × normal) often have poor outcomes, but peak total bilirubin is most predictive. A peak bilirubin >15 mg/dL (~256 μmol/L) is associated with ~4-fold higher odds of death. In contrast, extreme AST/ALT elevations alone do not independently predict mortality after adjustment. Thus, rising bilirubin during ECMO signals failing hepatic clearance and impending multi-organ failure.
- Renal Failure and CRRT: Acute kidney injury (AKI) requiring renal replacement therapy (CRRT) on ECMO is a grave prognostic sign. Historically, ECMO patients needing CRRT have mortality rates of around 80%. Contemporary series still show that concurrent RRT on ECMO confers significantly increased mortality risk (pooled ~60–70% mortality vs. ~40–50% without RRT). Although one study found CRRT itself did not raise mortality after adjusting for illness severity, the need for CRRT reflects severe multi-organ failure. It is an independent predictor of poor outcome on VA-ECMO (OR ≈1.2–1.8).
- Coagulation and Hematologic Factors: Systemic coagulopathy on ECMO often indicates clinical decline. An overt DIC (disseminated intravascular coagulation) score ≥5 within 24 h of ECMO initiation occurs in ~40% of patients and is associated with far higher mortality (55% vs. 36% without DIC). Patients with higher DIC scores (e.g., 7–8) suffer more bleeding and >80% mortality when combined with high lactate. Profound thrombocytopenia is also telling—a ≥ 50% drop in platelet count on Day 1 of VA-ECMO yields ~57% mortality versus 37% if platelets remain more stable. This significant early platelet decline carries an 8–9-fold increased odds of death, outperforming absolute platelet count as a prognostic marker. A prolonged INR (>1.5) frequently accompanies liver dysfunction and DIC in no survivors, reflecting loss of coagulation reserve. Likewise, extremely elevated D-dimer levels suggest ongoing coagulation/fibrinolysis (as in DIC or circuit thrombosis) and often parallel poor outcomes.
- Inflammatory Biomarkers: A severe inflammatory response on ECMO correlates with worse prognosis. Interleukin-6 (IL-6), a key cytokine, is often markedly elevated in patients with refractory shock or sepsis on ECMO; IL-6 levels above the median nearly tripled 90-day mortality in cardiogenic shock. Procalcitonin (PCT), indicating bacterial infection or systemic inflammation, likewise predicts outcome. Card Shock registry data showed PCT ≥ 0.5 μg/L associated with 50% mortality vs. 30% when PCT was lower. High IL-6 and PCT typically reflect an uncontrolled inflammatory cascade and tissue injury, whereas C-reactive protein (CRP), a nonspecific acute-phase marker, has less direct prognostic value in ECMO patients. Persistent elevation of IL-6 and PCT despite support suggests underlying sepsis or organ damage that may be irreversible.

## Introduction

Extracorporeal membrane oxygenation (ECMO) provides life-sustaining cardiac and respiratory support for patients in refractory cardiopulmonary failure. Advances in ECMO technology and critical care have expanded its use, yet mortality remains high—adult ECMO survival rates often hover around 40–60% (10). As illness severity escalates, patients on ECMO can develop progressive multi-organ failure despite maximal support. Clinicians then face difficult decisions on whether continued ECMO is futile and when to transition to palliative care. Identifying objective prognostic indicators is crucial to guide these ethical decisions and avoid both premature withdrawal and prolonged non-beneficial support. Along with the biomarker patterns already discussed, patient traits such as age, comorbidities, and neurological status are also essential when deciding whether to use ECMO.

A variety of blood and biochemical markers reflect the function (or failure) of major organ systems during ECMO. Liver enzymes and bilirubin track hepatic perfusion and function; renal output, creatinine levels, and need for continuous renal replacement therapy (CRRT) signal kidney injury; lactate levels and clearance gage systemic perfusion and metabolic stress; coagulation parameters (DIC score, platelets, INR, D-dimer) indicate hematologic disturbances; and inflammatory markers (IL-6, C-reactive protein, procalcitonin) reflect the severity of systemic inflammation or sepsis. Interpreting these biomarkers in tandem can help clinicians recognize when multi-organ failure is overwhelming and ECMO is unlikely to succeed.

We focus on how derangements in markers of each organ system correlate with outcomes, and how these data may inform interdisciplinary decisions regarding the continuation or cessation of ECMO support. We also propose an integrative approach to using these biomarkers to guide palliative transitions, emphasizing clear thresholds and trends that signal futility.

## Liver dysfunction markers: bilirubin and transaminases

Hepatic injury is common in ECMO due to shock, hypoxia, or congestion. However, not all liver enzymes carry equal prognostic weight. Notably, serum total bilirubin has emerged as a robust predictor of mortality in ECMO patients. In a 2022 single-center study of adult ECMO patients, peak total bilirubin levels were *twice as high* in 28-day non-survivors compared to survivors (median ~73 vs. 34 μmol/L) (2). A peak TBil >65 μmol/L (~3.8 mg/dL) was identified as an optimal cutoff predicting poor outcome (2). Patients exceeding this threshold had dramatically higher odds of death at 28 days (OR ~7.25) (2). The authors concluded that bilirubin elevation after ECMO initiation correlates with survival, “while other markers of liver injury do not” (2). In other words, hyperbilirubinemia appears to capture the cumulative impact of shock on the liver (e.g., impaired clearance, hemolysis, cholestasis) better than transient transaminase spikes.

Extensive cohort analyses support bilirubin’s prognostic value. In 152 ECMO patients over 9 years, the *highest* total bilirubin level [>15 mg/dL (≈256 μmol/L)] was associated with a 4.4-fold increase in the odds of in-hospital death (3). This was held in a multivariable model, whereas extreme ALT or AST elevations lost significance (3). Indeed, while 29–39% of patients had “shock liver” (AST or ALT >20 × upper normal) during ECMO (3), these acute enzyme surges did not independently predict mortality after adjusting for bilirubin and lactate levels (3). Peak bilirubin, in contrast, remained a significant predictor, echoing pediatric ECMO literature where TBil >15 mg/dL is a known high-risk indicator (3). The physiological basis is that bilirubin reflects not just hepatocellular injury but also the liver’s capacity to excrete bile and break down blood cells. A rising bilirubin often indicates worsening hepatic synthetic function and clearance, which are integrally linked to multi-organ failure.

Elevated bilirubin levels on ECMO also correlate with coagulopathy and the duration of support. Patients with high TBil tend to have prolonged prothrombin time, low fibrinogen, and a higher likelihood of failing to wean from ECMO (2). The of peak TBil above ~65 μmol/L had significantly higher mortality and more coagulopathic derangements than those below this threshold (2).

By contrast, transaminases (AST, ALT) primarily reflect acute hepatocellular injury, which may be severe but transient. Many ECMO patients experience drastic AST/ALT elevations (median peak AST ~ 420 U/L in one series) due to ischemic hepatitis or reperfusion injury (3). These indicate liver insult but are often self-limited if perfusion improves. Studies have found that although AST/ALT elevations correlate with mortality in univariate analysis, they frequently overlap with lactate and bilirubin as markers of shock severity (3). Once those factors are accounted for, transaminases per se are less prognostic (3). Clinically, an AST in the 1,000 signals severe shock liver, but a patient can still recover if other organ functions stabilize. In contrast, a bilirubin level climbing into the teens (mg/dL) suggests impaired hepatic clearance and ongoing hemolysis, often a later finding that heralds a low likelihood of recovery.

In summary, clinicians should view rising bilirubin on ECMO as a particularly ominous sign. A trend of bilirubin doubling or exceeding ~3–5 mg/dL warrants careful prognostication and discussion with the care team about futility, especially if accompanied by coagulopathy. Transaminase spikes underscore the severity of the initial insult but should be interpreted in context; if they do not resolve or if they coincide with bilirubinemia, the liver injury may be irreversible. Modern prognostic scores for ECMO, such as the SAVE score for VA-ECMO, include bilirubin ≥33 μmol/L (~1.9 mg/dL) as a component of “liver failure” in their risk stratification, underscoring the value of this marker (11, 12). In practice, persistent hyperbilirubinemia despite adequate circuit flow and oxygenation strongly suggests that multi-organ failure is progressing, and continued ECMO is unlikely to achieve recovery.

## Renal failure and ECMO: urine output, creatinine, and RRT

Renal dysfunction is exceedingly common in ECMO patients and is both a consequence and cause of poor outcomes. Acute kidney injury develops via multiple mechanisms on ECMO: low cardiac output and hypotension reduce renal perfusion; systemic inflammation and hemolysis can injure the kidneys; and aggressive diuresis or shock can lead to acute tubular necrosis. The practical hallmarks of severe renal failure are rising creatinine, oliguria (low urine output), and the need for renal replacement therapy. Each of these has prognostic significance.

Perhaps the starkest warning sign is the initiation of CRRT (continuous renal replacement therapy) during ECMO. Numerous studies document extremely high mortality in patients requiring dialysis while on ECMO support. Classic series reported mortality rates of 70–85% in ECMO patients who developed AKI and were started on CRRT (10). For instance, an observational study by Chen et al. noted an 85% hospital mortality in patients on combined ECMO+CRRT, versus ~58% overall mortality (10). Similarly, pediatric ECMO data showed that all children who needed hemofiltration had dramatically worse survival (10). These grim outcomes reflect that simultaneous severe cardiac/respiratory failure (necessitating ECMO) and kidney failure (necessitating CRRT) is a scenario of near-total organ collapse.

Reassuringly, recent meta-analyses suggest that outcomes have improved modestly over the last decade, but the prognosis remains poor. A 2021 systematic review pooled 24 studies (5,896 patients) and found an overall mortality of ~63% among ECMO patients receiving RRT (4). Notably, mortality in RRT-supported patients has decreased by about 20% in the most recent 5 years (post-2016), likely due to better patient selection and multidisciplinary care (4). Nonetheless, the analysis confirmed that the use of RRT on ECMO is associated with significantly higher mortality compared to ECMO patients not requiring RRT (relative risk ~1.8) (4). The need for dialysis thus remains an independent marker of critical illness severity. Consistently, multivariate analyses from large ECMO cohorts identify AKI requiring CRRT as an independent risk factor for death (6). In one center’s experience, the addition of CRRT was associated with a near doubling of mortality odds, even after adjustment for covariates (adjusted OR ~1.84) (4).

Interestingly, some studies that control for baseline severity have found that *CRRT itself* may not further worsen outcomes beyond what the patient’s condition already dictates. For example, Lamanna et al. examined 102 adult ECMO patients, half of whom received CRRT, and reported similar ICU mortality between the CRRT and non-CRRT groups (~60% vs. 46%, *p* = 0.23) (5). They concluded that CRRT was not associated with increased mortality in patients of similar characteristics (5). This suggests that the sickest patients often require CRRT, and their mortality is driven by overall severity rather than dialysis per se. Indeed, patients on CRRT had a higher incidence of immunosuppression and other comorbidities (5). Therefore, needing CRRT is more of a severity marker than a direct cause of death. Regardless, from a clinician’s standpoint, a patient on ECMO who progresses to oliguric renal failure requiring dialysis is in a critical state. It is rare for such patients to recover full organ function if other systems are also failing.

In daily practice, monitoring urine output is an immediate gage of renal perfusion. Oliguria (<0.5 mL/kg/h) or anuria on ECMO signals acute kidney injury and should prompt early nephrology consultation. The AKIN (Acute Kidney Injury Network) criteria can be applied even during ECMO; in fact, AKIN Stage 3 explicitly includes patients needing RRT (10). Studies using AKIN/RIFLE criteria on ECMO have shown that higher AKI stages correspond to progressively worse survival. For example, one report noted 100% mortality in patients meeting RIFLE-Failure criteria on day 1 of ECMO (10). Similarly, having AKIN Stage 3 AKI by 48 h on ECMO carried ~86% mortality (10). These scoring systems underscore that even the trajectory of creatinine and urine output in the first 1–2 days is predictive. If a patient on ECMO goes from normal renal function to dialysis-dependent within 48 h, it reflects catastrophic multi-organ failure.

Beyond survival, renal failure also impacts the quality of survival and resource use. Many ECMO survivors who had AKI can wean off dialysis over time, but some remain dialysis-dependent long-term (13, 14). The burden of CRRT adds complexity to ECMO management (anticoagulation, circuit connections, fluid balance) and may increase ICU length of stay for survivors (4). However, there is some evidence that proactive fluid removal with CRRT during ECMO could benefit survivors by preventing fluid overload (15). These nuances aside, the bottom line is that AKI development on ECMO is a pivotal turning point. Anuria and rising creatinine despite adequate flow suggest that perfusion is not reaching the kidneys, often a sign that even ECMO is insufficient to sustain end-organ perfusion.

From an ethical and palliative standpoint, the combination of ECMO and CRRT should trigger a re-evaluation of goals of care. If a patient has been on ECMO for several days with no renal recovery, requiring dialysis, and concurrently shows other markers of deterioration (e.g., high lactate, liver dysfunction), the likelihood of meaningful recovery is extremely low (4, 6). In such scenarios, interdisciplinary teams (intensivists, nephrologists, surgeons, and ethics consultants) should meet to discuss prognosis openly with the patient’s family. Many centers include “no improvement or worsening multi-organ failure” as a criterion in their ECMO withdrawal protocols. Renal failure is a key component of that. Indeed, one large ECMO registry analysis identified CRRT use, high DIC score, and age as independent predictors of mortality, suggesting these could form a bedside checklist for futility (6).

In summary, kidney markers on ECMO should be watched vigilantly. Lack of urine output and rising creatinine are early warning signs; if they progress to the need for CRRT, the care team should recognize this as an alarm bell. While modern techniques and improved ICU care have slightly attenuated the dire outcomes (mortality down from ~80% to ~60% in some series), the prognosis remains guarded (4). The presence of renal failure should significantly lower the threshold for considering ECMO discontinuation, especially if other organ systems concur that the patient is in refractory multi-organ failure.

## Markers of perfusion and metabolism: lactate and clearance

Serum lactate is one of the most critical real-time indicators of tissue perfusion in critically ill patients, including those on ECMO. Lactate is produced during anaerobic metabolism and as a stress response; thus, elevated lactate signifies global tissue hypoxia or severe adrenergic drive. In ECMO, clinicians closely monitor lactate trends as a gage of whether the patient’s perfusion is improving or worsening under support. High lactate levels before ECMO initiation often predict worse outcomes, and the evolution of lactate after ECMO initiation provides crucial prognostic information.

Extensive evidence links persistent hyperlactatemia to mortality. For example, a *post hoc* analysis of the HYPO-ECMO trial (VA-ECMO for cardiogenic shock) found that lactate levels were consistently higher at all-time points in non-survivors than in survivors (16). An initial lactate >2.2 mmol/L (upper limit of normal) approximately doubled the risk of 30-day mortality (HR 1.85) (16). Moreover, in that study, patients whose lactate failed to decrease over the first 7 days had poor outcomes, while survivors with high initial lactate showed a faster decline in lactate in the first 24 h (16). Notably, a secondary rise, or “rebound,” in lactate during ECMO, was a terrible prognostic sign: patients who experienced a second lactate increase had 1.78-fold higher hazard of death (16). This suggests that even if lactate initially improves, new complications (like sepsis or ischemia), causing lactate to climb again, are often lethal.

The prognostic power of lactate is evident as early as the first day on ECMO. A recent 2024 analysis of post-cardiotomy VA-ECMO patients demonstrated that absolute lactate levels at 12 and 24 h outperformed lactate clearance percentages in predicting mortality (1). Specifically, a 12-h lactate >8.2 mmol/L and a 24-h lactate >2.6 mmol/L were the best discriminators of eventual in-hospital death (AUROC 0.87–0.90) (1). These thresholds make clinical sense: by 24 h, a patient with adequate ECMO support and shock reversal should have lactate levels trending toward normal (<2–3 mmol/L). If lactate remains elevated above ~2.5 mmol/L at 24 h, it indicates significant tissue hypoxia or metabolic stress persists. On the other hand, lactate clearance—the percent decrease in lactate from baseline—also predicted outcomes, though slightly less robustly. Clearance <22% by 12 h or <40% by 24 h was associated with reduced survival and about a 2-fold increased hazard of death (1). In practical terms, if a patient’s lactate was, say, 10 mmol/L pre-ECMO and only dropped to 8 mmol/L at 12 h (20% clearance), that is worrisome. The cited study found that survivors tended to achieve greater lactate reductions (e.g., >40% by 24 h) than non-survivors (1). Kaplan–Meier curves illustrate a clear separation: patients with poor 12 h/24 h lactate clearance had significantly higher mortality (1).

It is worth noting that initial lactate before ECMO also matters, but less so than post-ECMO trends. Pre-ECMO lactate reflects how long and severe the patient’s shock was before support. For instance, an initial lactate >6 mmol/L was associated with increased mortality (AUROC ~0.73) in the 2024 study (1). However, many patients come to ECMO with very high lactate, yet if ECMO rapidly restores perfusion, lactate can clear, and the patient may survive. Thus, a high admission lactate is a red flag, but the trajectory under ECMO is more informative. Rapid lactate normalization is a positive sign; persistent or rising lactate despite ECMO suggests the support is either insufficient or the patient has irreversible pathology (e.g., massive cell death or mitochondrial dysfunction).

Beyond static thresholds, serial lactate monitoring provides a dynamic assessment. Studies have examined lactate at 6, 12, 24, and 48 h, among others. A common finding is that survivors achieve lactate decline within the first 24–48 h, whereas non-survivors often plateau or continue to rise. In a cohort of cardiogenic shock on VA-ECMO, serial daily lactates over the first week were all higher in non-survivors, and a secondary rise around day 3–4 portended near-certain mortality (16). Another study noted that peak lactate within 24 h was predictive—for example, peak 24 h lactate > ~ 10 mmol/L was associated with much higher 30-day mortality in an adult ECMO series (17). Lactate’s prognostic value is so established that it is included in ECMO outcome prediction scores. The ENCOURAGE score and SAVE score incorporate lactate (or lactate clearance) as key variables, acknowledging that a patient with refractory lactic acidosis on ECMO is unlikely to do well.

From a clinical management perspective, failure of lactate to improve should prompt reassessment of the ECMO strategy and the patient’s condition. If lactate remains high, one should ask: Is the circuit flow adequate? Is oxygen delivery sufficient (consider hemoglobin, oxygenator function)? Is there a hidden source of ischemia (e.g., compartment syndrome, limb ischemia, mesenteric ischemia)? Does the patient have profound sepsis or mitochondrial dysfunction that ECMO cannot fix? Often, a persistently elevated lactate indicates that despite normalizing microcirculatory parameters (blood pressure, cardiac output via ECMO), the microcirculation is not recovering—a classic sign of global tissue hypoperfusion and oxygen extraction deficit. This scenario might reflect irreversible shock or the need for adjunct therapies (e.g., vasodilators for microcirculation, improved venting of the left ventricle, etc.). If all correctable issues are addressed and lactate still will not clear, the prognosis is dismal.

Several high-profile studies exemplify lactate’s role: one analysis found that each 1 mmol/L increase in peak lactate was associated with a 15% increase in odds of death (3). In another study, patients with lactate >15 mmol/L had an extremely low survival (~10%) (17). Conversely, achieving lactate clearance >50% in 24 h correlated with better survival (~70%) (9). Lactate clearance has also been studied in ECPR (extracorporeal CPR after cardiac arrest); typically, if lactate does not decrease post-arrest, ECMO indicates catastrophic neurological and systemic damage, often leading to withdrawal of support.

In summary, lactate is arguably the single most useful biochemical marker for real-time prognostication on ECMO. It is readily available from blood gas analysis, responds quickly to changes in perfusion, and has well-defined cutoffs associated with outcome. Clinicians should define expectations for lactate improvement when initiating ECMO: e.g., “We expect the lactate to drop by half in the next 12 h if this is going to work.” If those expectations are not met, it is a strong signal to revisit the goals of care. When combined with markers of organ failure (such as the DIC score or bilirubin), lactate further enhances prognostic accuracy. In one septic shock ECMO study, a composite of pre-ECMO DIC score and lactate had an AUC of 0.88 for predicting hospital death—patients with a combined score above 9.35 had 82% mortality, versus 27% if below that score (7). This underscores how elevated lactate, together with coagulopathy, identifies a patient phenotype unlikely to survive.

Thus, for decision-making, if high lactate persists beyond 24–48 h on ECMO, especially in conjunction with other organ failures, clinicians should strongly consider it a marker of futility. At that juncture, families should be informed that “despite maximum support, the patient’s body is not recovering—the lactate (a marker of severe shock) remains very high, indicating organs are still starved of oxygen.” Such frank discussions, backed by lactate data, can facilitate consensus on transitioning to comfort-focused care when appropriate.

## Hematologic and coagulation markers: DIC, platelets, INR, D-dimer

Coagulation disturbances are ubiquitous in ECMO patients due to the combined effects of critical illness, inflammation, and contact of blood with the extracorporeal circuit. These disturbances range from manageable thrombocytopenia to fulminant disseminated intravascular coagulation (DIC). Because coagulation is so closely linked to inflammatory and organ failure pathways, markers of coagulopathy offer valuable prognostic insights. An overt DIC state or a precipitous drop in platelets often signals that the patient’s condition is deteriorating.

The International Society on Thrombosis and Hemostasis (ISTH) DIC score (based on platelet count, D-dimer/fibrin degradation products, prothrombin time, and fibrinogen) can be calculated in ECMO patients to identify overt DIC (score ≥5). Recent evidence indicates that early overt DIC in ECMO is a red flag for mortality. A 2024 study of 703 ECMO patients (both VA and VV) found that 24% met criteria for overt DIC within 24 h of ECMO initiation (6). Those with DIC had significantly higher in-hospital mortality (55% vs. 36% without DIC, *p* < 0.001) (6). Even successful weaning from ECMO was much less likely in the DIC group (only ~56% weaned, vs. 76% in the non-DIC group). Notably, DIC patients did not have markedly more thrombotic events overall in that study, but they did have more bleeding complications once the DIC score climbed very high (7, 8). The clear implication is that overt DIC reflects a profound loss of hemostatic control and microvascular thrombosis, which goes together with multi-organ failure. It is less about clinically evident clots or bleeding and more about the systemic derangement they represent.

Another investigation focusing on septic shock patients on ECMO showed that a high pre-ECMO DIC score correlates with poor outcome. In that cohort, combining DIC score with lactate (as mentioned earlier) sharply stratified mortality risk: patients with DIC + lactate score >9.35 had ~82% mortality vs. ~ 27% for lower scores (7). This underscores the synergy between coagulopathy and perfusion failure in driving outcomes. Mechanistically, severe infection or shock triggers uncontrolled coagulation (elevated D-dimer levels and consumption of clotting factors), which further impairs perfusion by forming micro clots in organs. Thus, once DIC sets in, it can accelerate the decline in organ function beyond what hypoperfusion alone would.

Clinically, what should one look for? Platelet count is a key component of the DIC score and an easily tracked lab. ECMO commonly causes thrombocytopenia due to platelet activation by non-endothelial surfaces and heparin use (8). A mild to moderate drop in platelets (to ~100–150 k/μL) is expected. However, an abrupt and/or severe drop is alarming. The magnitude of platelet decline in the first 24 h is particularly telling. A 2021 study of post-cardiotomy VA-ECMO patients demonstrated that those with a ≥ 50% relative drop in platelet counts on day 1 had far higher mortality (57%) than those with <50% drop (37%) (8). In multivariable analysis, a ≥ 50% decline in platelets was independently associated with a nearly 9-fold increase in the odds of in-hospital death (8). In fact, the AUC for predicting mortality was 0.78 for relative platelet drop, higher than that for absolute platelet count on day 1 (0.69) (8). This finding makes sense: a rapid crash in platelets signifies acute consumptive coagulopathy or severe platelet activation (e.g., due to massive SIRS or clot formation in the circuit). It is essentially an early DIC signal. On the other hand, a patient whose platelets remain relatively stable initially may have a better chance, barring other issues.

Platelet trends beyond day 1 also matter. Persistent thrombocytopenia (<50–80 k) and the requirement for frequent platelet transfusions suggest ongoing consumption, which often correlates with sepsis or DIC. Heparin-induced thrombocytopenia (HIT) is a special consideration on ECMO; HIT can cause precipitous platelet drops and thrombosis. But prognostically, a patient who develops HIT on ECMO is in danger both from thrombosis and from the difficulty of anticoagulation management. If HIT is confirmed, it adds another layer of complexity and risk.

INR (International Normalized Ratio) and fibrinogen are other pieces of the coagulopathy puzzle. A rising INR indicates loss of liver synthetic function or consumption of clotting factors, or both. In the critical care explorations study cited earlier, 70% of ECMO patients had an INR > 1.5 at some point (3). While INR > 1.5 was associated with mortality on univariate analysis (unadjusted OR ~3.65), it did not remain independently predictive after accounting for bilirubin and lactate (3). This again highlights how coagulation abnormalities often travel with liver dysfunction and shock. Nonetheless, an INR that is climbing despite vitamin K and plasma support is a grave sign, usually indicating fulminant liver failure or DIC. Low fibrinogen (<1 g/L) on ECMO also suggests advanced DIC (since fibrinogen is consumed) and often necessitates cryoprecipitate transfusions. These measures can temporize bleeding risk, but if the underlying process (sepsis, trauma, malignancy) driving DIC is not resolving, outcomes remain poor.

D-dimer, a fibrin degradation product, is typically very elevated in ECMO patients. Even at baseline, ECMO circuits activate coagulation and fibrinolysis, elevating D-dimer levels. Extremely high D-dimer levels (e.g., >10–20 μg/mL) are characteristic of DIC. One study noted that D-dimer is part of the best prognostic model in septic ECMO (the DIC + Lactate model) (7). In practice, D-dimer trends can be hard to interpret because ECMO-related thrombosis (microthrombi in the oxygenator or filters) can also elevate D-dimer levels. However, if D-dimer is increasing exponentially and the patient has clinical DIC, it only reinforces the severity of the situation. Some centers monitor antithrombin III (AT III) levels as well, since AT III gets consumed in DIC and with heparin therapy. The 2024 study found that AT III levels were inversely correlated with DIC score (*r* = −0.417) and that low AT III levels had a decent predictive value for overt DIC (c-statistic 0.81 for AT III to detect DIC) (6). Severe AT III deficiency was associated with higher mortality (49% vs. 34%). This suggests that AT III could be a surrogate marker of coagulopathy severity.

In summary, coagulation markers add prognostic context to the ECMO patient’s trajectory. A practical approach: calculate an ISTH DIC score daily for ECMO patients, especially those who are septic or have liver impairment. If the DIC score is rising or ≥5, be aware that the mortality risk is climbing (6). Watch the platelet count trend closely; a halving of platelets overnight should prompt a search for causes (HIT, occult clotting, DIC) and merit prognostic caution (8). Observe the INR and fibrinogen; if these are deranging, they often mirror the severity of liver dysfunction and inflammatory consumption. Consider repeating the DIC score and lactate together—as noted, the combination is potent for gaging futility. A high DIC score indicates the patient’s coagulation system is “giving up,” which is often accompanied by or precedes other organs giving up.

Finally, coagulopathy has direct ramifications for ECMO management: patients in DIC are at high risk of both bleeding (e.g., intracranial hemorrhage) and thrombosis (circuit clots). Sometimes, development of DIC or uncontrollable bleeding forces the clinician’s hand to stop ECMO because it is no longer safe (for example, massive hemorrhage or inability to maintain circuit flow due to clots). In an ethical sense, that scenario is both a complication and a prognosis—if the patient has bled into the brain or has clotted off the circuit due to DIC, the chances of meaningful recovery are exceedingly low. Thus, DIC can be both a cause and a marker of futility. The goal is to recognize the trajectory toward DIC early, using the lab trends discussed, so that decisions can be made proactively rather than reactively in the face of catastrophe.

## Inflammatory biomarkers: IL-6, CRP, and Procalcitonin

Critical illness and ECMO trigger a vigorous inflammatory response, often termed “cytokine storm” in severe cases. Inflammation and organ failure are intertwined—excessive cytokines can drive capillary leak, myocardial depression, coagulation activation, and more. Tracking inflammatory biomarkers such as interleukins, C-reactive protein (CRP), and procalcitonin (PCT) can provide insight into the patient’s systemic inflammatory burden and underlying infections. These markers are not organ-specific, but their extremes can indicate a patient who is “too sick” to recover despite support.

Interleukin-6 (IL-6) is a key pro-inflammatory cytokine frequently elevated in sepsis, cytokine release syndromes, and severe tissue injury. In ECMO patients, IL-6 levels tend to be very high if the patient’s underlying condition is septic shock, major trauma, or severe infection (such as COVID-19 ARDS). High IL-6 levels have been associated with worse outcomes. For example, in a multi-center study of cardiogenic shock patients (CardShock study), those with IL-6 levels above the median had a 90-day mortality of 57% vs. 22% for those below the median (9). This was a significant difference (*p* < 0.01), and IL-6 was one of the strongest predictors of outcome in that cohort (9). While that study was not exclusively ECMO, many cardiogenic shock patients were supported with devices, and it illustrates IL-6’s prognostic gravity. The same study found that IL-6 > median was associated with all the hallmarks of worse shock: higher lactate levels, greater acidosis, and greater organ dysfunction (9). Essentially, IL-6 served as a barometer of shock severity and was tightly linked to mortality.

In ECMO-specific contexts, IL-6 is also notable. Reports from the COVID-19 pandemic (where many patients were placed on VV-ECMO for ARDS) observed that patients with persistently elevated IL-6 fared poorly, often dying or having prolonged courses (18, 19). Some small studies found that IL-6 levels before ECMO initiation could predict outcome in COVID-19; one noted that each log increase in IL-6 was associated with higher odds of death, or that IL-6 above a certain threshold increased mortality risk by a measurable percentage (19). Moreover, cytokine filtration or hemoadsorption has been attempted on ECMO circuits to remove IL-6 and other cytokines, with the hypothesis that reducing cytokine load might improve outcomes. While the results are inconclusive, the need for cytokine adsorption suggests the patient has a severe hyperinflammatory state.

C-reactive protein (CRP) is a general marker of inflammation produced by the liver. It rises in response to infection and tissue injury, but is slower and less specific than cytokines like IL-6. In critical illness, CRP often peaks a couple of days into the course. Interestingly, the CardShock study found that CRP_max was not prognostically significant in their cohort (9). CRP did rise in all patients (median peak ~137 mg/L), but survivors and non-survivors had overlapping CRP ranges (9). This suggests that while CRP confirms the presence of inflammation, it does not differentiate the degree of shock as well as IL-6 or PCT. In ECMO patients, CRP tends to be uniformly high due to surgical cannulation, any underlying infection, and the inflammatory response to extracorporeal circulation. A failure of CRP to decrease after many days might indicate ongoing infection. Still, acute CRP values are not very helpful to prognosticate survival, especially compared to dynamic markers like lactate or IL-6.

Procalcitonin (PCT) is a peptide precursor of calcitonin that rises dramatically in bacterial infections and sepsis. It also rises in systemic inflammation (even sterile) to some extent but is regarded as more specific for infection than CRP. PCT kinetics is faster; levels can climb within hours of insult and fall with resolution or appropriate antibiotic therapy. In shock states, high PCT levels are associated with organ failure severity and infection burden. The CardShock study mentioned above found that 60% of patients had PCT_max ≥0.5 μg/L, and these patients had significantly worse profiles (more hypoperfusion, higher IL-6, more renal failure) (9). Their 90-day mortality was 50%, compared to 30% in those with PCT_max <0.5 μg/L (9). This indicates a relative risk of ~1.7 for mortality with high PCT (20). Notably, PCT and IL-6 often correlated—patients with high PCT frequently had high IL-6, both reflecting severe shock with a likely infectious or inflammatory cause (9). In that study, PCT and IL-6 were prognostic, whereas CRP was not, highlighting PCT’s usefulness.

In ECMO patients, one specific scenario is secondary infections (ventilator-associated pneumonia, bloodstream infection, etc.) that can complicate the course. A rising PCT on ECMO might alert clinicians to a new infection. From a prognosis standpoint, an ECMO patient who is *both* in cardiopulmonary failure and has overwhelming sepsis (marked by very high PCT, e.g.,>10 μg/L) is far less likely to survive than a patient on ECMO for isolated cardiac failure without sepsis. PCT can help quantify that. For instance, neonatal studies in congenital diaphragmatic hernia (CDH) on ECMO showed that extremely high PCT levels were linked with non-survivable outcomes (21). High PCT in neonates likely indicates uncontrolled infection or bowel ischemia. In adults, PCT > 5–10 μg/L on ECMO often means septic shock on top of primary failure.

Another inflammatory marker sometimes monitored is ferritin, especially during COVID-19, as a marker of macrophage activation or HLH-like hyperinflammation. Very high ferritin (e.g., >2,000 ng/mL) might indicate a cytokine storm, which, in the context of ECMO, could mean the patient has a severe inflammatory syndrome (e.g., MIS-C in children or secondary HLH). While not explicitly asked in the prompt, it is worth noting that if IL-6 and other markers are off-the-charts, ferritin probably is too, which paints a picture of a patient with almost unresolvable inflammation.

In practical terms, how to use these inflammatory markers? If IL-6 is available, a significantly elevated IL-6 (hundreds to thousands of pg./mL) should temper expectations of recovery. Some centers consider IL-6 > 1,000 pg./mL as a sign of cytokine storm. Suppose such levels persist or worsen despite therapy (e.g., appropriate antibiotics, source control, immunomodulation). In that case, it indicates that the patient’s immune response is in a “fulminant” state that ECMO cannot resolve. CRP can be trended, but often is not the make-or-break data point for prognosis. PCT is appropriate because it ties into infection control—if PCT remains high, either the infection is not controlled or the patient is still in severe sepsis-induced organ dysfunction. From a decision standpoint, a patient on ECMO with, say, PCT 50 μg/L, IL-65,000 pg./mL, and DIC is almost certainly not going to survive; this could guide clinicians to recommend withdrawal.

It is essential, however, to interpret inflammatory markers in context. For example, IL-6 might spike after a one-time event like surgery, then decrease—that is less concerning than an IL-6 that starts high and stays high or keeps rising. Also, therapies can modulate these markers (e.g., tocilizumab, an IL-6 blocker, reduces IL-6 levels, but does not mean the patient is better—it just blocks the receptor). So, the trend is key. A consistent failure of CRP or PCT to fall over a week on ECMO when expected (assuming infections are being treated) suggests ongoing inflammatory drive, which often correlates with poor outcome.

In summary, inflammatory biomarkers reflect the “background fire” that might be consuming the patient beyond the primary cardiac or respiratory failure. When they are significantly elevated or remain so despite treatment, they add to the evidence that continuing life support may be futile. These markers support a holistic view: for instance, combining an IL-6 of 1,000 pg./mL with a lactate of 10 mmol/L and a DIC score of 6 clearly paints a dire picture. Conversely, if a patient’s inflammatory markers normalize (CRP downtrending, PCT clearing) while on ECMO, it is a positive sign that sepsis or inflammation is under control, allowing organs to recover. Thus, the trajectory of these markers should inform daily rounds and family meetings about how the patient is truly doing beyond just the pump flow and ventilator settings.

## Integrating multi-system markers for prognosis and ethical decision-making

Each biomarker category—hepatic, renal, perfusion, coagulative, and inflammatory—provides a distinct “window” into the patient’s ECMO status. Moreover, neurological outcomes (including serious brain injury) constitute a vital prognostic factor that must be included with biomarker patterns in the assessment of futility. The challenge for clinicians is to integrate these myriad data points into a coherent assessment of whether the patient is improving or irreversibly deteriorating. No single lab value should be used in isolation; rather, trends and combinations are key. In practice, experts often consider scores or comprehensive models of multiple organ markers to decide on futility.

One approach is to use formal scoring systems. The Sequential Organ Failure Assessment (SOFA) score, for example, incorporates bilirubin, creatinine (or urine output), platelets, hypotension, etc., to quantify multi-organ failure. A high or rising SOFA score on ECMO is strongly associated with mortality (1). Indeed, survivors in one study had a median SOFA of 9 at ECMO initiation, vs. 13.5 in non-survivors (1). Tracking SOFA can summarize in a single number what is happening across organs—if it is not decreasing under ECMO support, the trajectory is poor. Another specialized score, the SAVE score for VA-ECMO, includes variables such as age, organ failures (liver, renal, CNS), lactate, and cardiac arrest, and provides a predicted survival probability. A very low SAVE score (e.g., ≦ 10) implies <20% expected survival (22). Such prognostic scores can be presented to ethics committees or families to provide an objective framing (e.g., “Given his clinical profile, his predicted chance of survival to discharge is only ~10% by this validated model”). However, scores are imperfect and should not replace clinical judgment; they should complement the bedside impression gleaned from all the lab trends discussed.

Increasingly, the concept of “ECMO futility” is being defined by multi-organ criteria. The Extracorporeal Life Support Organization (ELSO) guidelines encourage members to consider withdrawal of ECMO when there is “no hope for survival” or unacceptable neurological injury. Many centers have adopted specific triggers, such as “No *signs of cardiac recovery by day X, AND evidence of severe brain injury OR multiple organ failure* (e.g.*, MELD >a threshold, or requirement of >2 organ supports*).” In this context, the markers we discussed are essential for defining “multiple organ failure.” For instance, one could operationalize a policy that ECMO should be discontinued if three or more of the following occur: lactate >10 mmol/L for >12 h, bilirubin >10 mg/dL and rising, on CRRT >7 days with no renal output, DIC with ISTH score ≥5 and uncontrolled bleeding, and presence of sepsis with PCT > 10 μg/L despite antibiotics. While each institution’s criteria differ, the principle remains the same: combine markers to determine whether the patient has crossed the point of no return.

Another real-world example is the use of time limits in combination with markers. Some programs say, “We will support on ECMO for up to 2 weeks. By day 10, if the patient has shown *no* improvement in markers (e.g., lactate clearance, improving hepatic/renal labs) and has worsening labs, we will initiate end-of-life discussions.” This time-limited ECMO trial can be very helpful in setting expectations from the start. Families can be told: “We will try ECMO for a period, but if during that time we see markers indicating things are getting worse, we might have to stop.” It is easier for families to accept withdrawal if they have been prepared for certain lab criteria that would guide the decision. It also lends an aura of objectivity—that the decision is based on the patient’s condition, not on whim.

Interdisciplinary decision-making is critical. An ECMO patient often receives input from intensivists, surgeons, cardiologists, nephrologists, and neurologists. Each specialty might focus on its domain markers (e.g., neurologists on brain CT results and sedation needs, nephrologists on BUN/Cr and fluid balance). Regular meetings or huddles should be held to consolidate these perspectives. Ethics consultations are valuable when the path is unclear—they can help weigh the burdens vs. benefits of continued ECMO, given the patient’s values and medical indicators.

Communication of prognostic markers to the patient’s family is a delicate art. Clinicians should use understandable terms: for example, “His liver and kidneys are shutting down—the bilirubin number that measures liver function has kept rising, and he has not made urine even with the dialysis machine. His blood acid level (lactate) remains very high, which tells us his body is not getting better despite the ECMO. We worry that continuing this is only prolonging the dying process.” Families appreciate transparency and education; often, showing trends on a whiteboard (e.g., a downward arrow for platelets, an upward arrow for bilirubin and lactate) can help illustrate the point. It is also important to stress the irreversibility aspect—that if these numbers indicated potentially fixable problems, the team would persist, but unfortunately, they indicate overwhelming failure.

One must also consider patient-centered outcomes, such as neurological status, in prognostication If a patient has had prolonged low flow or anoxic injury (perhaps reflected indirectly in high lactate and DIC, suggesting no perfusion to the brain for a period) or other devastating neurological complications (e.g., large strokes), the neurological prognosis could be grim and tilt the balance toward non-recovery, even if other organs recover. This ties into decisions because a patient surviving ECMO only to be in a vegetative state is generally not a desired outcome; in fact, current ECMO guidelines include severe brain injury as a criterion for withdrawal of support, underscoring its significance. Thus, neurological exams and imaging should complement laboratory tests to provide a complete prognostic picture. However, the scenario described in this review is one of systemic failure, in which the brain is also usually at risk.

As we integrate these markers, a pattern emerges: the worst-case scenario ECMO patient will demonstrate a confluence of deranged markers—e.g., lactate soaring, bilirubin accumulating, no urine output/CRRT, DIC with falling platelets and rising D-dimer, and high IL-6/PCT from relentless inflammation. Such a profile is almost universally fatal. On the flip side, a patient trending in the right direction will show lactate clearance, stable or improving end-organ labs, and controlled inflammatory markers, often within the first 3–5 days of ECMO. This divergence in trajectories typically becomes evident within the first week, which is usually when decisions about continuing vs. withdrawing ECMO are made. Some authors have suggested an “ECMO Multi-Organ Dysfunction Score” or adapting the SOFA daily to quantify progress (6). Future research may refine these into bedside decision aids.

Table 1 summarizes the key prognostic biomarkers identified in recent studies, along with threshold values and associated outcomes.

**Table 1:** Key ECMO prognostic biomarkers and their implications.

Biomarker or parameter	Prognostic threshold/sign	Implications for outcome
Lactate (perfusion)	>8 mmol/L at 12 h, or >2.5 mmol/L at 24 h; poor clearance (<22% reduction in 12 h).	Strongly predicts high mortality; failure to clear lactate early is an early warning of futility. (Example: lactate >8 at 12 h or persistent lactic acidosis was associated with very low survival.)
Total bilirubin (hepatic)	Peak >15 mg/dL (≈256 μmol/L) during ECMO.	~4-fold higher odds of death if exceeded; rising bilirubin indicates shock liver and failing hepatic clearance, often heralding multi-organ failure.
Acute kidney injury requiring CRRT	Need for dialysis (CRRT) during ECMO support.	Grave prognostic sign—historically ~80% mortality; in recent studies ~60–70% mortality with CRRT vs. ~ 40–50% without. Indicates severe multi-organ failure (CRRT requirement is an independent predictor of mortality).
DIC score (coagulation)	ISTH DIC score ≥5 within 24 h of ECMO initiation.	Early overt DIC is a red flag— ~ 55% mortality vs. 36% if no DIC. If combined with high lactate, it indicates an inferior prognosis (e.g., DIC + lactate score >9.3 → ~82% mortality).
Platelet count drop	≥50% decline in platelets within the first 24 h.	Signifies consumptive coagulopathy; associated with ~57% mortality vs. 37% if no drop, and ~8–9 × higher odds of death. (An abrupt early platelet crash is essentially an early DIC signal of physiologic decline.)
Inflammatory markers (e.g., IL-6, PCT)	Extremely high levels (IL-6 in the thousands of pg./mL; PCT > 10 μg/L) or persistent elevation despite treatment	Signals cytokine storm or uncontrollable sepsis. For example, IL-6 > 1,000 pg./mL is considered a feeble prognostic sign; an adult on ECMO with IL-6 ~ 5,000 pg./mL and PCT ~ 50 μg/L is almost invariably fatal. Such extreme or unremitting inflammation correlates with a near-zero chance of survival.

It is crucial to emphasize that the prognostic thresholds analyzed in this review are derived from heterogeneous studies across different ECMO populations. People who have cardiogenic shock or septic shock, or newborns who have respiratory failure, may act in various ways. These biomarkers and cut-offs are helpful, but you should be wary of relying on them too much. When it comes to interpreting these signals for each patient, clinical judgment remains the most essential factor.

In conclusion, biochemical markers spanning all major organ systems are invaluable for guiding ECMO management and end-of-life decisions. Whether they improve or worsen over time, the direction of these markers is just as important. These trends give us considerably more helpful information about the future than any one lab number. They provide objective evidence of whether a patient is recovering or in extremis, helping clinicians avoid both prolonged futile support and premature cessation. High-quality, compassionate care in ECMO includes not only state-of-the-art life support but also knowing when to say “enough.” The data from liver, kidney, perfusion, coagulation, and inflammatory markers, taken together, empower clinicians and families to make that determination with clarity and confidence. Such decisions should always be made through a multidisciplinary deliberation—involving intensivists, surgeons, neurologists, ethicists, and others—to ensure that all perspectives (including the patient’s age, comorbidities, and neurological status) are considered alongside biomarker evidence before transitioning to palliative care. As ECMO use grows and ethical dilemmas become more common, ongoing research (especially in the 2015–2025 era) continues to refine these prognostic indicators (3, 4). Ultimately, integrating these lessons into clinical protocols will ensure that ECMO is deployed not only as a heroic lifesaver but also withdrawn with wisdom and dignity when the limits of medicine are reached.
